# Benzoxaborole-Based Inhibitors Block LexA Autocleavage and Suppress SOS-Dependent Adaptive Phenotypes in *Escherichia coli*

**DOI:** 10.3390/antibiotics15050437

**Published:** 2026-04-27

**Authors:** Pierangelo Bellio, Lisaurora Nazzicone, Lorenza Fagnani, Eleonora Scarsella, Donatella Tondi, Laura Bertarini, Giuseppe Celenza

**Affiliations:** 1 Department of Biotechnological and Applied Clinical Sciences, University of L’Aquila, 67100 L’Aquila, Italy; pierangelo.bellio@univaq.it (P.B.); lisaurora.nazzicone@graduate.univaq.it (L.N.); eleonora.scarsella@graduate.univaq.it (E.S.); giuseppe.celenza@univaq.it (G.C.); 2 Department of Life Sciences, University of Modena and Reggio Emilia, Via Campi 103, 41125 Modena, Italy; donatella.tondi@unimore.it (D.T.); laura.bertarini@unimore.it (L.B.)

**Keywords:** antimicrobial resistance, SOS response, LexA, RecA, benzoxaboroles, drug repurposing, bacterial adaptation, biofilm formation

## Abstract

**Background/Objectives**: The rapid emergence of antimicrobial resistance (AMR) is driven not only by antibiotic selective pressure but also by bacterial adaptive responses that enhance genetic diversification under stress. The SOS response, regulated by the RecA-LexA axis, plays a central role in coordinating DNA repair, mutagenesis, and phenotypic adaptation. Targeting this pathway represents a promising strategy to limit bacterial adaptability without directly affecting viability. This study aimed to evaluate benzoxaborole-based compounds as potential inhibitors of the LexA regulatory pathway. **Methods**: A drug repurposing approach was employed to investigate the benzoxaborole scaffold and the clinically approved derivatives tavaborole and crisaborole. Biochemical assays were used to assess LexA autocleavage in a RecA-dependent co-protease system. Molecular docking analyses were performed to evaluate compound binding within the LexA catalytic site. Microbiological assays were conducted to examine the effects on antibiotic-induced filamentation and biofilm formation under different growth conditions. **Results**: Selected benzoxaboroles inhibited LexA autocleavage, with tavaborole showing the strongest inhibitory profile in the biochemical assay. Docking analyses supported these findings, indicating stable binding within the LexA catalytic site near the catalytic serine residue. At the cellular level, tavaborole and benzoxaborole significantly reduced levofloxacin-induced filamentation at sub-inhibitory concentrations. Both compounds also decreased biofilm formation under nutrient-limited conditions, while no significant effects were observed on preformed biofilms. Crisaborole showed limited cellular activity despite measurable biochemical effects. **Conclusions**: These findings identify benzoxaboroles as modulators of the LexA-dependent SOS response and support the potential repurposing of clinically approved compounds as adjuvants to limit bacterial adaptive responses associated with antimicrobial resistance.

## 1. Introduction

The escalation of antimicrobial resistance (AMR) is one of the most pressing challenges to global health, with projections estimating 10 million annual deaths by 2050 if current trends persist [1]. While traditional antibiotic discovery has focused on identifying compounds that directly kill bacteria or inhibit their growth, this “biocidal” pressure inevitably selects for resistant mutants. Consequently, a paradigm shift is underway towards “anti-evolutionary” therapies: strategies designed not merely to kill pathogens but to disable their ability to adapt and evolve resistance under stress [2,3].

Resistance commonly arises through genetic adaptation, including mutations in antibiotic targets and the acquisition of foreign DNA via horizontal gene transfer (HGT), which facilitates the rapid spread of resistance genes [4]. These mechanisms, compounded by the widespread misuse of antibiotics in both clinical and veterinary medicine, generate strong selective pressure that promotes the emergence of multidrug-resistant (MDR) pathogens [5], thereby substantially complicating antimicrobial therapy. Addressing this crisis requires not only the development of new antibiotics but also the identification of alternative therapeutic targets. Among potential new targets, the RecA-LexA pathway has gained attention for its role in bacterial stress responses, stress adaptation, and the evolution of antibiotic resistance. In this context, this study adopts a drug-repurposing approach to identify boron-containing compounds that inhibit LexA autocleavage, thereby blocking activation of the SOS response.

The SOS response is a global bacterial regulatory network activated by DNA damage and replication stress, involving over 60 genes and primarily controlled by the RecA-LexA regulatory axis. 

However, the activation of the SOS response is not exclusively associated with DNA repair [6]. The expression of DNA-damage-inducible genes also contributes to diverse bacterial phenotypes, including antibiotic resistance gene transfer [7], persistence and subpopulation tolerance [8], adhesion and biofilm formation [9,10], filamentation [3], and motility changes [10].

The SOS response is regulated by two key proteins: LexA, a transcriptional repressor, and RecA, a DNA-damage sensor [11]. LexA represses the expression of numerous genes involved in various cellular processes, including regulation of cell division and DNA repair. This regulation is mediated by the specific binding of the LexA N-terminal domain to the lexA box, also known as the SOS box, a 16–20 bp palindromic DNA motif with the consensus sequence 5′-TACTGTATATATATACAGTA-3′ located near the RNA polymerase binding site [12,13,14,15].

RecA serves as the central molecular switch that governs activation of the SOS response. It belongs to the recombinase family and is highly conserved across the vast majority of bacterial species [16,17]. Functional homologs of RecA have also been identified in bacteriophages [18], archaea [19,20,21] and eukaryotic cells [22,23,24,25,26].

Through its ability to sense DNA damage and replication stress, RecA initiates the molecular cascade that leads to activation of the SOS response. DNA damage or replication fork stalling serves as the activating signal, causing RecA to bind single-stranded DNA and form an activated nucleoprotein filament that promotes LexA self-cleavage [11,27].

Given its pivotal role in regulating bacterial stress responses, LexA is a promising and unconventional target for antimicrobial intervention. Inhibiting its autoproteolytic activity would prevent activation of the SOS response and reduce the emergence of resistant phenotypes.

Given that LexA self-cleavage is serine-dependent and that boron-containing compounds are well-established serine-protease inhibitors [28,29], our approach was to identify boron-based molecules with potential inhibitory activity against the transcriptional repressor under investigation. Specifically, this choice stems from evidence obtained by our group [3], which first demonstrated that small boron-containing molecules can effectively inhibit the self-cleavage of the transcriptional repressor LexA. These findings were subsequently corroborated by independent data from Chatterjee et al. [30], further validating this strategy.

In the search for effective LexA inhibitors, boron-containing compounds, particularly benzoxaboroles, have emerged as a “privileged scaffold” in medicinal chemistry. The empty p-orbital of the boron atom enables these molecules to form reversible covalent adducts with nucleophilic residues, such as serine or threonine hydroxyls, in the active sites of various enzymes [31]. This mechanism is validated by two FDA-approved drugs: Tavaborole (AN2690), a topical antifungal that targets fungal leucyl-tRNA synthetase (LeuRS) via a boron-tRNA adduct [32], and Crisaborole (AN2728), a topical phosphodiesterase-4 (PDE4) inhibitor for atopic dermatitis [33].

Beyond their primary target, benzoxaboroles have shown broader pharmacological potential, including antibacterial activity and synergy with antibiotics [34,35].

Building on our previous identification of boron-based LexA inhibitors [3], this study investigates the potential of Tavaborole and Crisaborole to be repurposed as modulators of the *E. coli* SOS response. By combining biochemical assays, molecular docking, and phenotypic screens (filamentation and biofilm inhibition), we aim to determine whether these clinical-stage benzoxaboroles can effectively target the bacterial SOS master regulator.

## 2. Results

### 2.1. Co-Protease Activity and Determination of Half-Maximal Inhibitory Concentrations (IC_50_)

**Figure 1:**
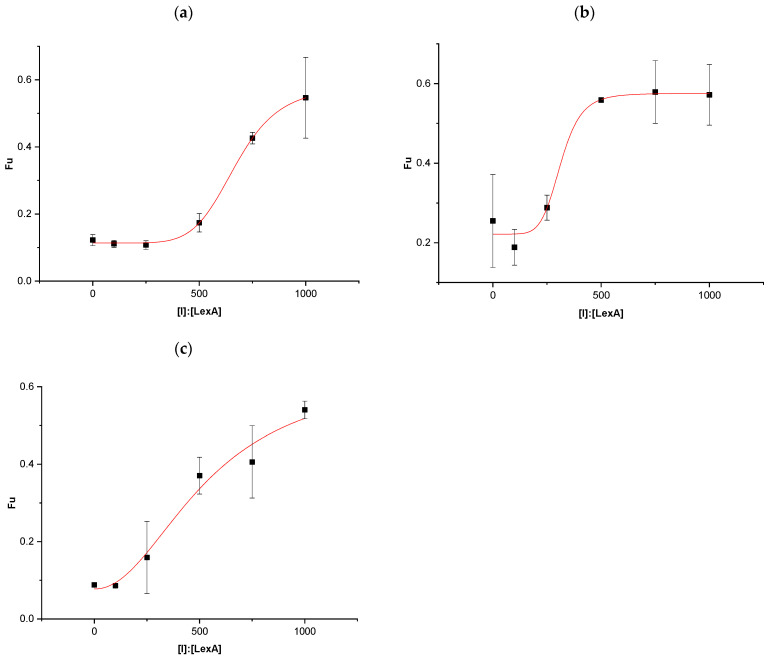
Plots of uncleaved LexA fraction (Fu, *Y*-axis) versus the inhibitor concentration, expressed as molar ratios ([I]:[LexA], *X*-axis) for 1-hydroxy-3H-2,1-benzoxaborole (**a**), tavaborole (**b**), and crisaborole (**c**).

To quantitatively compare the inhibitory potency of the three boron-containing compounds, half-maximal inhibitory concentrations were determined by measuring RecA-LexA co-protease activity. Under these conditions, the fraction of uncleaved LexA was evaluated as a function of inhibitor concentration, expressed as molar ratios of inhibitor to LexA ([I]:[LexA]). The resulting dose–response curves are reported in Figure 1.

The calculated IC_50_ values were 671.7 ± 48 for 1-hydroxy-3H-2,1-benzoxaborole (Figure 1a), 313.07 ± 70.6 for tavaborole (Figure 1b), and 538.8 ± 245.2 for crisaborole (Figure 1c). When expressed as absolute concentrations, these values corresponded to 8.33 mM, 3.88 mM, and 6.68 mM, respectively.

### 2.2. Determination of the Minimum Inhibitory Concentrations (MICs)

To define the concentration ranges to be employed in subsequent filamentation and biofilm assays, the minimum inhibitory concentrations of the three benzoxaboroles were determined for *E. coli* BL21(DE3) and *E. coli* ATCC^®^ 25922 using the broth microdilution method. In addition, the MIC of levofloxacin was determined exclusively in *E. coli* BL21(DE3), as this strain was used for filamentation assays in which levofloxacin served as the inducing agent. MIC values for both strains are reported in Table 1.

Both 1-hydroxy-3H-2,1-benzoxaborole and tavaborole displayed measurable inhibitory activity against the two *E. coli* strains, with MIC values ranging from 8 to 16 µg/mL. In contrast, crisaborole did not exhibit inhibitory activity within the tested concentration range, showing MIC values greater than 128 µg/mL for both strains. Among the tested benzoxaboroles, tavaborole exhibited the lowest MIC values.

### 2.3. Filamentation Assay

**Figure 2:**
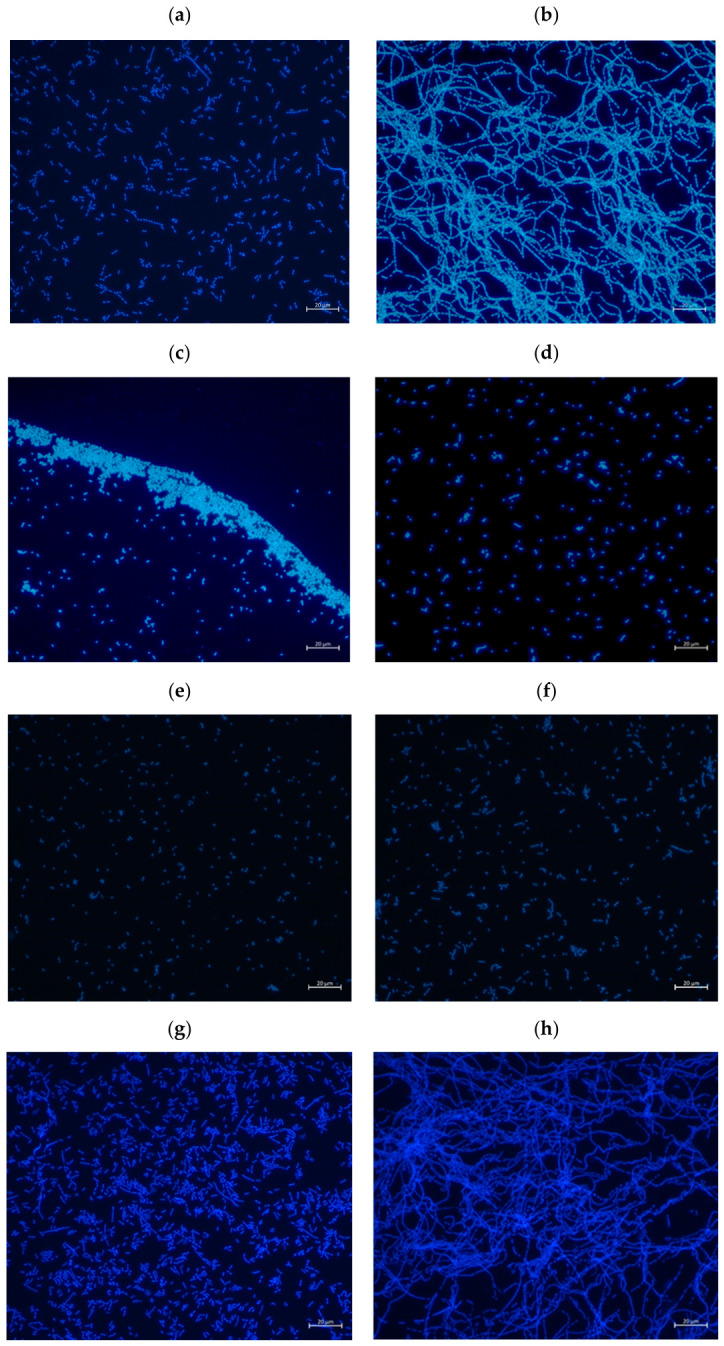
Fluorescence microscopy of *E. coli* BL21(DE3) (DAPI staining, 40×). (**a**) Untreated control; (**b**) levofloxacin (1.9 × 10^−3^ µg/mL); (**c**) 1-hydroxy-3H-2,1-benzoxaborole (16 µg/mL); (**d**) 1-hydroxy-3H-2,1-benzoxaborole (16 µg/mL) + levofloxacin (1.9 × 10^−3^ µg/mL); (**e**) tavaborole (8 µg/mL); (**f**) tavaborole (8 µg/mL) + levofloxacin (1.9 × 10^−3^ µg/mL); (**g**) crisaborole (128 µg/mL); (**h**) crisaborole (128 µg/mL) + levofloxacin (1.9 × 10^−3^ µg/mL). The scale bar corresponds to 20 µm.

Since filamentation represents a well-established phenotypic hallmark of SOS activation, the effect of the boron-containing compounds on levofloxacin-induced filamentation was evaluated in *E. coli* BL21(DE3). As shown in Figure 2, exposure to levofloxacin alone induced pronounced filamentation (Figure 2b) compared to untreated control cells (Figure 2a). When tested individually, none of the boron-containing compounds induced filamentation (Figure 2c,e,g).

In combination with levofloxacin, 1-hydroxy-3H-2,1-benzoxaborole and tavaborole completely suppressed filament formation (Figure 2d,f). In contrast, the combination of levofloxacin with crisaborole resulted in only a partial reduction in filamentation (Figure 2h), with filamentous structures still detectable, although reduced in number and length compared to levofloxacin treatment alone.

**Figure 3:**
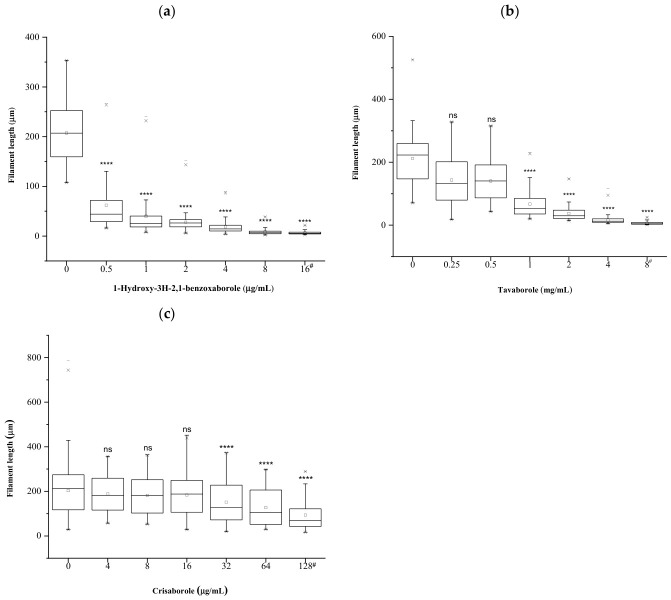
Plots of the mean filament length as a function of boronic compound concentration. Filament length is expressed relative to levofloxacin-treated cells. (**a**) 1-hydroxy-3H-2,1-benzoxaborole; (**b**) tavaborole; (**c**) crisaborole. ^#^ MIC value; **** *p* < 0.001; ns, not significant.

Quantitative analysis of filament length confirmed the qualitative observations reported above (Figure 3). Filament length was measured and expressed as the percentage reduction relative to levofloxacin-treated cells alone (Tables S1–S3). Both 1-hydroxy-3H-2,1-benzoxaborole and tavaborole significantly inhibited levofloxacin-induced filamentation at all tested concentrations, displaying a clear concentration-dependent effect (Figure 3a,b). In contrast, crisaborole reduced filamentation only at the three highest concentrations tested, while no appreciable effect was observed at lower concentrations (Figure 3c and Figures S1–S6).

### 2.4. Biofilm Assay: Inhibition and Eradication

**Figure 4:**
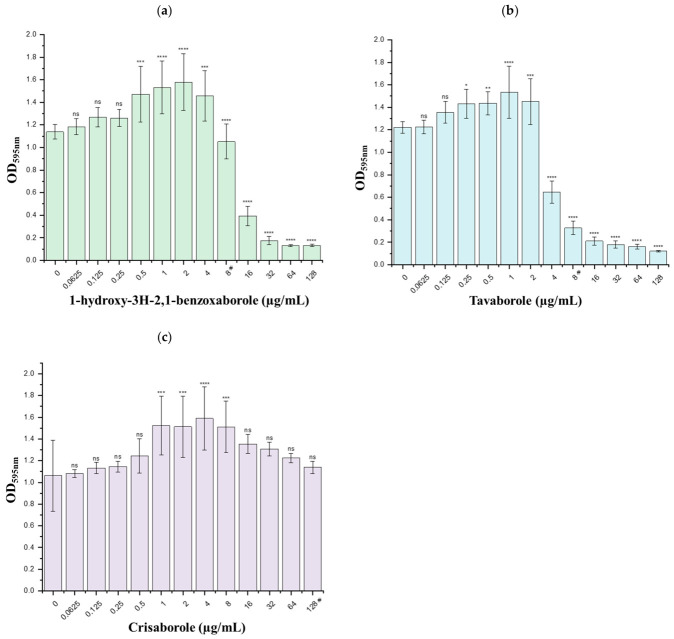
Plots of the mean biofilm biomass formed in the presence of the tested boronic compounds. (**a**) 1-hydroxy-3H-2,1-benzoxaborole; (**b**) tavaborole; (**c**) crisaborole. **^#^** MIC value; * *p* < 0.05; ** *p* < 0.01; *** *p* < 0.005; **** *p* < 0.001; ns, not significant.

Given the involvement of the SOS response in the early stages of biofilm development, biofilm formation was assessed in *E. coli* ATCC^®^ 25922 under nutrient-limiting conditions. Under these conditions, a significant inhibitory effect on biofilm formation was observed for both 1-hydroxy-3H-2,1-benzoxaborole (Figure 4a) and tavaborole (Figure 4b), whereas crisaborole did not display detectable inhibitory activity (Figure 4c). Biofilm inhibition was quantified as the percentage reduction relative to untreated control biofilms, with detailed values reported in Tables S4–S6.

For 1-hydroxy-3H-2,1-benzoxaborole, concentrations corresponding to 2 × MIC, 4 × MIC, 8 × MIC, and 16 × MIC resulted in a marked reduction in biofilm biomass, with inhibition values ranging from 65% to 88%. Tavaborole showed inhibitory effects over a broader concentration range, extending from 0.5 × MIC to 16 × MIC, and produced biofilm reductions between 47% and 90%. In contrast, crisaborole did not significantly affect biofilm formation at any of the concentrations tested, and higher concentrations could not be evaluated due to poor solubility in the culture medium.

To determine whether these compounds were also able to affect established biofilms, biofilm eradication assays were performed on preformed biofilms. As shown in Figure S7, none of the boron-containing compounds exhibited a substantial eradication effect, as biofilm biomass remained comparable to untreated controls at all tested concentrations.

### 2.5. Molecular Docking

To provide a structural description of the interactions between the boronic compounds and LexA, molecular docking simulations were performed to analyse the binding orientations adopted within the active site, with particular attention to the region surrounding the catalytic Ser119 residue. Two main binding orientations were identified and defined according to the position of the free hydroxyl group relative to key residues lining the binding pocket: an “upward” conformation, in which the hydroxyl group is oriented toward Thr154, Val155, and Lys156, and a “downward” conformation, in which it is directed toward the catalytic core, involving Gly117, Met118, Ser119, and Met120.

**Figure 5:**
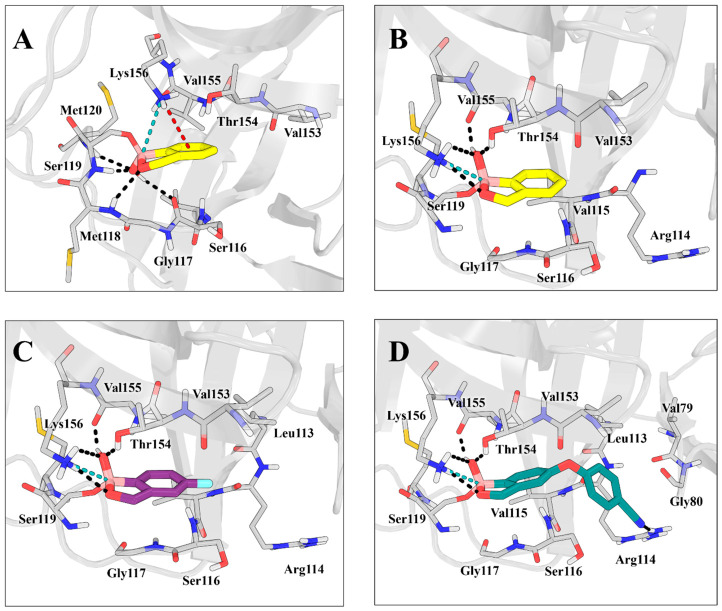
Docking poses of the selected compounds within the LexA binding site (PDB ID: 1jhf). The protein is depicted as a grey cartoon, with the residues lining the binding pocket shown as capped sticks. Key interactions are indicated by dashed lines: hydrogen bonds in black, cation–π interactions in red, and salt bridges in teal. The “downward” orientation of benzoxaborole (yellow) is shown in (**A**), while (**B**) illustrates its “upward” orientation. Docking poses of tavaborole (purple) and crisaborole (deep teal) are presented in (**C**,**D**), respectively. All images were generated using PyMOL version 3.1.6.1.

For unsubstituted benzoxaborole, both binding orientations were observed. In the “downward” conformation (Figure 5A), the free hydroxyl group is oriented toward the catalytic region and forms hydrogen bonds with the backbone carbonyl and amide groups of Ser116, Ser119, and Met120, while the ring oxygen engages in hydrogen bonding with the backbone amide of Met118. In this configuration, the boron atom forms a salt bridge with the side chain of Lys156, and a stabilizing cation–π interaction is observed between the aromatic ring and the ε-ammonium group of Lys156.

In the alternative “upward” conformation (Figure 5B), the salt bridge between the tetrahedral boron and Lys156 is maintained, although the overall geometry differs. In this orientation, the ring oxygen is directed toward Lys156 and forms a hydrogen bond with its ε-amino group, while the free hydroxyl group participates in a hydrogen-bonding network involving Lys156, the backbone carbonyl of Val155, and the β-hydroxyl group of Thr154.

Overall, the “upward” conformation exhibited more favourable docking scores (−4.154 kcal/mol) and MM-GBSA binding free energies (−27.80 kcal/mol), consistent with a more stable and well-defined interaction network within the catalytic region. Conversely, the “downward” conformation was less conserved across docking poses and was associated with less favourable docking scores and MM-GBSA values (−3.954 kcal/mol and −9.68 kcal/mole, respectively), reflecting reduced stabilization of the complex in this orientation.

Substitution on the phenyl ring resulted in a marked preference for the upward-facing orientation. In the case of tavaborole, the presence of a fluorine substituent enhanced interactions with Lys156, Val155, and Thr154, contributing to stabilisation of the complex (Figure 5C). Notably, the predicted binding poses for tavaborole were highly conserved and largely superimposable, indicating a well-defined and stable binding mode within the active site. In contrast, for the bulkier derivative crisaborole, steric effects similarly favoured the “upward” orientation but led to a broader distribution of poses, reflecting increased conformational variability. This resulted in less conserved binding geometries, lower docking scores and more diverse orientations within the pocket, while still enabling additional stabilizing interactions, most notably between the cyano group and Arg114 (Figure 5D). Consistent with the structural analysis described above, docking scores and MM-GBSA binding free energies are reported in the Supplementary Information (Table S7). Notably, tavaborole exhibited the most favourable docking score (−5.053 kcal/mol), whereas crisaborole showed the least favourable value (−3.820 kcal/mol); accordingly, docking results remain consistent with the IC_50_ trend (tavaborole > 1-hydroxy-3H-2,1-benzoxaborole > crisaborole). In contrast, MM-GBSA values do not fully reproduce this order, likely reflecting the limitations of endpoint free energy approximations in capturing the full complexity of the system.

## 3. Discussion

Antimicrobial resistance (AMR) is increasingly recognised as a phenomenon driven by evolutionary adaptation rather than purely being a pharmacological failure. Consequently, therapeutic strategies designed to modulate bacterial adaptability, by interfering with stress-response and mutagenesis pathways, have emerged as promising anti-virulence approaches that complement traditional antibiotics [36,37]. Within this context, the transcriptional repressor LexA constitutes an unconventional yet strategically significant target. Its inhibition is not aimed at directly impairing bacterial viability, but rather at preventing the activation of the SOS response, thereby decreasing the selective pressure that contributes to resistance development.

The present study expands upon our prior identification of small boron-containing molecules as inhibitors of LexA autoproteolysis [3]. Building on this conceptual foundation, we employed a rational assessment strategy to evaluate benzoxaborole scaffolds, including compounds already in clinical use, to determine their potential as modulators of bacterial adaptability rather than as conventional antibacterial agents. The strategy of interfering with LexA cleavage by RecA has recently been further validated by approaches beyond small-molecule inhibition. Notably, Maso et al. [38] described the development of nanobodies derived from camelid heavy-chain antibodies that bind LexA with sub-micromolar affinity. Structural analysis revealed that these nanobodies inhibit the SOS response by stabilising LexA in a conformation incompetent for cleavage and physically blocking its interaction with the RecA nucleoprotein filament [38,39]. While our benzoxaboroles exploit a small-molecule covalent mechanism targeting the catalytic serine, the efficacy of these macromolecular inhibitors confirms that “locking” LexA in a non-cleavable state is a robust method to suppress bacterial adaptation.

Accurate interpretation of biochemical data necessitates consideration of the intrinsic characteristics of the target. Unlike canonical Michaelian enzymes, LexA undergoes a single-turnover autoproteolytic reaction, during which the catalytic event is accompanied by irreversible functional inactivation of the protein [11]. In this context, inhibition should not be viewed as the blockade of a repetitive catalytic cycle but rather as the modulation of the proportion of functionally available LexA molecules. Within this framework, the millimolar IC_50_ values observed in the RecA-LexA co-protease assay (approximately 3.8 mM for tavaborole and 8.3 mM for 1-hydroxy-3H-2,1-benzoxaborole) should not be interpreted as indicative of weak intrinsic activity. A direct comparison between biochemical and cellular data reveals an apparent discrepancy between these millimolar IC_50_ values and the micromolar concentrations effective in phenotypic assays. For example, tavaborole showed activity at 8–16 µg/mL (approximately 50–100 µM), representing a >50-fold difference relative to its biochemical IC_50_ (~3.8 mM). As previously discussed, the most meaningful measure of potency for LexA inhibitors is the inhibitor-LexA molar ratio [3]. Instead, the observed values reflect the regulatory nature of the target: for a transcriptional repressor such as LexA, even partial modulation may hold biological significance if it is sufficient to attenuate downstream adaptive responses [40].

The adoption of the RecA-LexA co-protease system further supports this interpretation. This assay recapitulates the physiological interaction between the DNA damage sensor RecA and the repressor LexA, thereby providing a context that closely mimics in vivo SOS activation. In this setting, benzoxaboroles maintained the same potency ranking observed in preliminary experiments (tavaborole > 1-hydroxy-3H-2,1-benzoxaborole > crisaborole), consistent with direct target engagement and in line with docking predictions.

To further rationalise the biochemical and phenotypic observations, molecular docking simulations were conducted to examine the binding orientations of boronic compounds within the LexA active site, with particular focus on interactions near the catalytic Ser119 residue. Boron-containing derivatives are recognised to engage with serine hydrolases via the formation of an anionic tetrahedral intermediate, whereby the boron atom establishes a covalent bond with the nucleophilic hydroxyl group of the catalytic serine, accompanied by a hybridisation change from planar *sp*^2^ to tetrahedral *sp*^3^ [3,41,42]. This interaction mode aligns with the catalytic mechanism underlying LexA autoproteolysis and lends support to the hypothesis of boron-based inhibition.

Docking analyses demonstrated that substitutions on the phenyl ring significantly affect conformational preferences within the active site, promoting an upward-facing binding orientation. In this configuration, a conserved interaction network involving Lys156 is consistently maintained: the oxygen atom of the benzoxaborole ring forms a hydrogen bond with the ε-amino group of Lys156, while the tetrahedral boron centre creates a stabilising salt bridge with the same residue. Collectively, these interactions enhance binding stability and ensure proper positioning of the covalent adduct.

Although the parent benzoxaborole scaffold can adopt both upward- and downward-facing conformations, aromatic substitution appears to bias more substituted analogues towards the upward-facing geometry. This conformational constraint provides a structural rationale for the preserved biochemical activity observed across derivatives and suggests that side-chain modifications primarily influence orientation and stability within the binding pocket rather than abolishing target engagement. At the same time, these structural variations significantly affect physicochemical properties relevant to intracellular accumulation, offering a coherent explanation for the dissociation observed between biochemical potency and whole-cell activity, particularly in the case of crisaborole.

Nevertheless, biochemical inhibition alone does not suffice to validate a LexA-targeting strategy. Translation of target engagement into cellular phenotypes remains essential. The filamentation assay, therefore, represents a critical bridge between biochemical activity and cellular response, serving both as a canonical readout of SOS modulation and as an indirect indicator of intracellular target accessibility [43]. Phenotypic readouts such as filamentation, while informative, are not exclusive indicators of SOS activation and may be influenced by additional regulatory pathways; therefore, the observed effects should be interpreted in the context of the combined biochemical and phenotypic evidence. Tavaborole and 1-hydroxy-3H-2,1-benzoxaborole effectively suppressed levofloxacin-induced filamentation at sub-inhibitory concentrations, whereas crisaborole displayed limited cellular efficacy despite demonstrable biochemical activity. This dissociation does not challenge the validity of LexA as a target; rather, it underscores the permeability constraints imposed by the Gram-negative outer and inner membranes [44]. The bulky cyanophenyl substituent and associated physicochemical properties of crisaborole likely restrict intracellular accumulation, thereby limiting effective target engagement despite favourable in silico binding.

This permeability-driven divergence highlights a central principle: inhibition of LexA is a necessary but not sufficient condition for phenotypic modulation. Adequate physicochemical characteristics that enable intracellular access are equally critical. In this respect, tavaborole, originally optimised for transungual penetration, appears to offer a more favourable balance of size, polarity, and permeability [45], enabling effective modulation of SOS-associated phenotypes in whole cells.

It is important to note that a direct correlation between LexA inhibition and minimum inhibitory concentration (MIC) values should not be expected. LexA is a non-essential regulator under standard growth conditions; consequently, selective inhibition of LexA is not anticipated to produce bactericidal effects [12]. The antimicrobial activity observed with tavaborole (MIC 8–16 µg/mL) is therefore unlikely to result solely from SOS inhibition. Tavaborole is a well-characterised inhibitor of leucyl-tRNA synthetase (LeuRS), functioning through the formation of a boron–tRNA adduct that disrupts protein synthesis [32]. The antibacterial activity observed in this context most plausibly reflects a dual-targeting mechanism, combining the inhibition of an essential enzyme (LeuRS) with modulation of a global regulatory network (LexA).

Such polypharmacological behaviour aligns with the classification of benzoxaboroles as “privileged scaffolds” that engage multiple serine- or hydroxyl-rich targets [31]. From an evolutionary standpoint, simultaneous targeting of essential and regulatory pathways may impose a fitness cost that is difficult to overcome through single-point mutations, aligning with therapeutic strategies that limit resistance rather than maximise immediate bactericidal potency. At the same time, this broad target engagement may influence selectivity and should be taken into account in the further development of these compounds.

The functional consequences of SOS modulation are further supported by biofilm assays. The SOS response plays a key role in initiating biofilm formation but is less involved in maintaining mature biofilm structures [9,10]. Consistent with this model, tavaborole significantly inhibited biofilm formation, with up to a 90% reduction, while showing no activity against preformed biofilms. A modest increase in biofilm formation observed at sub-inhibitory concentrations may reflect a stress-induced adaptive response. Such behaviour is consistent with a hormetic-like effect, whereby low concentrations of antimicrobial agents can transiently stimulate biofilm formation as a protective strategy, although this effect was not specifically investigated in the present study.

Rather than representing a limitation, this temporal specificity reinforces the mechanistic coherence of LexA modulation and distinguishes benzoxaboroles from non-specific biofilm-disrupting agents.

Finally, suppression of levofloxacin-induced filamentation at sub-inhibitory concentrations highlights the potential anti-evolutionary role of these compounds. Unlike DNA-damaging antibiotics that trigger SOS-mediated filamentation and mutagenesis, benzoxaboroles directly interfere with LexA cleavage, thereby preventing the derepression of error-prone polymerases such as Pol V, which are central drivers of stress-induced mutagenesis [2,46]. In this light, recent observations that tavaborole synergises with aminoglycosides to suppress resistance emergence [34] are mechanistically consistent with the data presented here.

In conclusion, this study does not propose benzoxaboroles as novel antibiotics but rather identifies them as modulators of bacterial adaptability. By targeting the RecA-LexA axis, these compounds act on the regulatory machinery underlying stress-induced evolution. The availability of clinically approved molecules such as tavaborole, together with coherent biochemical, cellular, and phenotypic evidence, supports their potential as therapeutic adjuvants aimed at modulating bacterial adaptability responses.

## 4. Materials and Methods

### 4.1. Organisms

*E. coli* BL21(DE3) with genotype F- *ompT gal dcm lon hsdSb(rb−mb−) λ*(DE3 [*lacI lacUV5-t7p07 ind1 sam7 nin5*]) [*malB+*]_k−12_(λs) was purchased from Sigma-Aldrich (Milan, Italy), and *E. coli* ATCC^®^ 25922 came from the American Type Culture Collection (ATCC, Manassas, VA, USA).

*E. coli* BL21(DE3) was used for filamentation assays, as this B-lineage strain, characterised by reduced protease activity, may favour increased persistence of SOS regulators such as SulA, thereby enhancing filamentation responses. In contrast, ATCC^®^ 25922 was employed for biofilm experiments, as it is a widely used reference strain for antimicrobial susceptibility testing according to CLSI guidelines and represents a robust and reproducible model for biofilm formation assays.

### 4.2. Antibiotic and Boron-Based Compounds

The antibiotic levofloxacin (LVX) was purchased from Sigma-Aldrich (Milan, Italy). 1-Hydroxy-3H-2,1-benzoxaborole, tavaborole and crisaborole (Table 2) were from Vinci-Biochem (Florence, Italy).

### 4.3. Reagents

Luria–Bertani (LB) broth, Mueller-Hinton (MH) broth, Dulbecco’s phosphate-buffered saline (PBS), Tris-HCl, dithiothreitol (DTT), glycine, potassium phosphate buffer (KPB, 0.1 M), sodium chloride (NaCl), magnesium chloride (MgCl_2_), dimethyl Sulfoxide (DMSO), Coomassie Brilliant Blue R250, crystal violet (0.1% *w*/*v*), β-mercaptoethanol, Fluoroshield™ mounting medium containing DAPI (4′,6-diamidino-2-phenylindole), paraformaldehyde, glutaraldehyde, adenosine triphosphate (ATP), acetone, ethanol, hexamethyldisilazane (HMDS) were purchased from Merck (Darmstadt, Germany). Laemmli sample buffer (4×) and Mini-PROTEAN^®^ Precast Gel were obtained from Bio-Rad (Milan, Italy). Poly(dT)_36_ was purchased from Eurofins Genomics (Ebersberg, Germany). M63 minimal medium was prepared in-house according to the standard formulation. BIOMOL^®^ Green reagent was purchased from Enzo Life Sciences (Farmingdale, NY, USA).

### 4.4. RecA and LexA Cloning and Purification

RecA and LexA proteins were cloned and purified as previously described [3,47]. Briefly, the *recA* and *lexA* genes, encoding for RecA and LexA, respectively, were cloned separately into pET28b(+) expression vector. Recombinant plasmids were transformed in *E. coli* JM109 (DE3) competent cells [genotype *endA1*, *recA1*, *gyrA96*, *thi*, *hsdR17* (*rk^−^*, *mk^+^*), *relA1*, *supE44*, *λ–*, *Δ(lac-proAB)*, [*F’*, *traD36*, *proAB*, *lacI^q^ZΔM15*], λDE3] for proteins expression.

RecA was purified using two HisTrap FF 1 mL columns in series, whereas LexA purification was performed using a single HiTrap FF column. The proteins were quantified by the Bradford assay, and protein purity was assessed by SDS-PAGE.

### 4.5. Co-Protease Activity and Determination of Half-Maximal Inhibitory Concentrations (IC_50_)

The IC_50_ values of the boron-containing compounds were determined by evaluating the co-protease activity RecA-LexA. LexA concentration was fixed at 12.4 µM and increasing inhibitor-LexA molar ratios (0:1, 100:1, 250:1, 500:1, 750:1, 1000:1) were tested. To activate RecA (1.29 µM) and induce LexA self-cleavage, reactions were supplemented with 5.74 µM poly(dt)_36_ and 500 µM ATP. Inhibitors were first pre-incubated with LexA at 30 °C for 15 min, and subsequently the reaction was initiated by the addition of ATP and conducted at 30 °C for 10 min in assay buffer (20 mM Tris-HCl pH 7.5, 8 mM MgCl2, 1 mM DTT). Reactions were stopped by adding 4× Laemmli Sample Buffer supplemented with β-mercaptoehanol at a final concentration of 2.36 M, and samples processed as described above. The IC_50_ values were calculated from the residual fraction of uncleaved LexA obtained from three independent experiments.

Control experiments were performed to evaluate whether the compounds directly inhibited RecA ATPase activity. Each boron-containing compound was individually tested on RecA in the absence of LexA using the Biomol Green ATPase assay under identical buffer conditions. No inhibition of RecA ATPase activity was observed for any of the tested compounds.

Samples were denatured by boiling at 100 °C for 7 min, separated on Mini-PROTEAN^®^ Precast Gel (Bio-Rad, Milan, Italy) by SDS-PAGE, and stained with Coomassie Brilliant Blue R250 (Merck, Darmstadt, Germany). After destaining, gels were visualised using a ChemiDoc XRS+ System (Bio-Rad, Hercules, CA, USA). The uncleaved LexA fraction was quantified by densitometric analysis using ImageLab^TM^ 6.1 software (Bio-Rad) and expressed as the mean residual fraction obtained from three independent experiments.

The residual fraction of uncleaved LexA (*Fu*) was calculated using the following equation:

(1) $$Fu=\frac{\frac{{LexA}_{I}}{{RecA}_{I}}}{\frac{{LexA}_{C-}}{{RecA}_{C-}}}$$

where *LexA_I_* and *RecA_I_* represent the residual LexA and RecA in treated samples, respectively; *LexA_C−_* and *RecA_C−_* correspond to the negative controls.

IC_50_ values were determined by nonlinear regression analysis using a four-parameter logistic model implemented in OriginPro 2018 SR1 (OriginLab, Northampton, MA, USA). Goodness-of-fit was assessed by chi-squared value and the adjusted coefficient of determination (R^2^).

### 4.6. Determination of the Minimum Inhibitory Concentrations (MICs)

The in vitro antimicrobial effect of levofloxacin and boron-containing compounds was evaluated using the broth microdilution method in U-bottom 96-well polystyrene microtiter plates, as previously described [5]. MIC values were determined in accordance with Clinical and Laboratory Standards Institute (CLSI) [48]. Bacterial growth in each well was quantified spectrophotometrically at 595 nm using a Beckman Coulter (Brea, CA, USA) DTX-880 (Multimode Detector) microplate reader. The percentage growth was calculated using the following equation:

(2) $$\frac{{OD}_{drug\;containing\;well}-{OD}_{background}}{{OD}_{drug\;free\;well}-{OD}_{background}}$$

where background corresponds to microorganism-free wells. MIC values were defined as the concentration of the drug that reduces growth by 80% compared to the growth of microorganisms grown in the absence of the drug. MICs were reported as the median of three independent experiments.

### 4.7. Filamentation Assay

The filamentation assay was performed using *E. coli* BL21(DE3). Filamentation was induced by LVX at a subinhibitory concentration of 1.9 × 10^−3^ µg/mL (1/16 × MIC). Boron-containing molecules, at concentrations ranging from MIC to 1/32 × MIC, were tested in combination with LVX. The assay was performed as previously described [3] with minor modifications. Briefly, 500 μL of LB broth were inoculated with an overnight culture of *E. coli* BL21(DE3) to obtain a final bacterial concentration of 1 × 10^6^ CFU/mL and supplemented with LVX, boronic compounds, or drug-free control. Cultures were incubated at 37 °C for 3 h on an orbital shaker. Cultures were centrifuged at 14,000 rpm for 10 min at 4 °C and washed with PBS. Cell pellets were then resuspended in 4% (*v*/*v*) paraformaldehyde prepared in PBS and incubated on ice for 30 min. After removal of the fixative by washing with PBS, pellets were resuspended in 100 μL of PBS, spotted onto poly-L-lysine-coated glass slides and air-dried overnight at room temperature. Slides were washed with deionised water and mounted with Fluoroshield™ containing DAPI. Filamentation was visualised by fluorescence microscopy and quantified using ImageJ software as the percentage reduction in filament length relative to cultures treated with levofloxacin alone. For each experimental condition, measurements were obtained from at least three independent replicates. Within each replicate, approximately 50–150 individual filaments were analysed, depending on cell density and image quality.

Statistical analysis was performed using GraphPad Prism software version 5.0 (San Diego, CA, USA). Data normality was assessed using the Shapiro–Wilk test and found to deviate from a normal distribution. Differences among groups were evaluated using the non-parametric Kruskal–Wallis test, followed by Dunn’s multiple comparisons test for post hoc analysis.

Only comparisons with the levofloxacin-treated condition were considered. Statistical significance was set at *p* < 0.05, and significance levels were reported as **** *p* < 0.001.

### 4.8. Biofilm Assay

Biofilm formation was evaluated using the method described by Merritt et al. [49], with minor modifications. *E. coli* ATCC^®^ 25922 was used for biofilm experiments. All boron-containing compounds were dissolved in DMSO, and the final DMSO concentration in all assays did not exceed 1% (*v*/*v*). Equivalent solvent-only controls were included in all experiments. Bacterial cultures were grown overnight in LB broth at 37 °C under aerobic conditions in an orbital shaker, then subcultured for approximately 2 h under the same conditions. Upon reaching an optical density at 600 nm of ~0.4, the culture was diluted 1:10 in M63 minimal medium and dispensed into flat-bottom 96-well polystyrene microtiter plates, yielding a final bacterial concentration of 3 × 107 CFU/mL. Plates were incubated for 24 h at 37 °C under static conditions. Planktonic growth was assessed by measuring OD_595nm_ using a Beckman Coulter DTX-880 (Multimode Detector) microplate reader. Non-adherent cells were then removed, and wells were gently washed with deionised water. Biofilms were heat-fixed at 60 °C for 1 h, stained with 0.1% (*w*/*v*) crystal violet, washed thoroughly with deionised water, and air-dried overnight at room temperature. For quantitative analysis, crystal violet was solubilised using an ethanol/acetone solution (80:20, *v*/*v*) for 15 min, and OD_595nm_ was measured. Biofilm formation was calculated using the following equation:

(3) BF=AB−CW

where *BF* represents biofilm formation, *AB* corresponds to the OD_595nm_ of stained attached bacteria, and *CW* is the OD_595nm_ of stained control wells containing medium only [50].

#### 4.8.1. Biofilm Inhibition Assay

For the biofilm inhibition assay, each boron-containing compound was tested at concentrations ranging from 128 µg/mL to 0.0625 µg/mL, with six replicates per concentration. After bacterial inoculation, compounds were added to the wells, and plates were incubated at 37 °C for 24 h under static conditions, following the same experimental setup as the biofilm formation assay. Biofilm biomass was quantified, and results were expressed as the percentage reduction in biofilm formation relative to inhibitor-free control biofilms (Tables S4–S6). Each experiment was repeated three times independently.

#### 4.8.2. Biofilm Eradication Assay

For the biofilm eradication assay, boronic compounds were added to preformed 24 h mature biofilms and incubated for an additional 96 h at 37 °C under static conditions [51]. Each compound was tested at concentrations ranging from 128 µg/mL to 0.0625 µg/mL, with six replicates per concentration, following the same experimental setup as above. Residual biofilm biomass was quantified, and results were expressed as the percentage of preformed biofilm eradicated relative to inhibitor-free control biofilms. All experiments were independently repeated three times.

### 4.9. Molecular Modelling

Molecular docking studies were conducted for selected benzoxaborole derivatives using the Maestro interface (Schrödinger LLC, version 14.4.133, New York, NY, USA). The high-resolution crystal structure of LexA in its apo form (PDB ID: 1JHF) was used as the receptor model for all simulations. Prior to docking, the protein structure was processed using the Protein Preparation Wizard, which included assigning bond orders, adding hydrogens, optimising hydrogen-bonding networks, and predicting protonation states at physiological pH (7.4) using PROPKA. The structure was then minimised using the OPLS4 force field, with convergence criteria set to a heavy-atom RMSD of 0.30 Å. All crystallographic water molecules and ions were removed. To evaluate the druggability of the LexA binding site, SiteMap was used to identify and score potential pockets across the protein structure. Ligands were prepared using LigPrep with Epik, generating three-dimensional structures of the selected benzoxaborole derivatives and optimising their protonation states at pH 7.0 ± 0.5. For covalent docking of boronic acid derivatives, Ser119 was designated as the reactive residue, modelling the boronic acid addition reaction mechanism. The docking grid was centred on Ser119 and its neighbouring residues Ser116, Thr154 and Lys156. Covalent docking was performed using the CovDock module, specifically designed for modelling covalent interactions. Finally, MM-GBSA calculations were conducted to estimate binding free energies and provide further insights into ligand–receptor binding affinity.

### 4.10. Software and Data Analysis

Gel acquisition and band quantification were obtained using ImageLab^TM^ 6.1 software. Filament lengths were measured with ImageJ 1.53t. Data analysis and non-linear fitting were carried out using OriginPro 2018 SR1 (OriginLab).

## 5. Conclusions

The transcriptional repressor LexA represents a promising and unconventional antimicrobial target, given its pivotal role in orchestrating bacterial stress response and its widespread conservation across microbial species. Despite organism-specific variations, the SOS response governs conserved adaptive pathways in many bacteria. Crucially, the absence of eukaryotic homologs makes LexA an attractive target for selective inhibition, minimising the risk of host-associated toxicity.

Within this framework, boron-based compounds represent a rational and innovative class of inhibitors. Their favourable safety profile, combined with their ability to interfere with serine-dependent autoproteolytic mechanisms, positions boronic acids as well-suited molecules for targeting LexA function. Our results identify benzoxaboroles as a promising chemical scaffold that can modulate LexA activity depending on the substituent. Notably, tavaborole and crisaborole, both clinically approved drugs, demonstrated measurable interaction with LexA, highlighting their potential for drug repurposing. This strategy offers a significant translational advantage by accelerating development timelines while relying on established safety and pharmacovigilance data.

Beyond direct interference with the SOS response, boronic acids have also been reported to impair biofilm formation, a major contributor to antimicrobial resistance and treatment failure. Collectively, these findings underscore the versatility of boron-containing compounds and support their continued investigation as multifunctional agents aimed at modulating antimicrobial resistance through non-traditional therapeutic strategies.

## Figures and Tables

**Table 1:** MIC values of benzoxaboroles against both *E. coli* BL21(DE3) and *E. coli* ATCC^®^ 25922.

Compound	MIC Value (µg/mL)	
	*E. coli* BL21 (DE3)	*E. coli* ATCC^®^ 25922
Levofloxacin	3.1 × 10^−2^	-
1-hydroxy-3H-2,1-benzoxaborole	16	8
Tavaborole	8	8
Crisaborole	>128	>128

**Table 2:** Boron-based compounds included in the study.

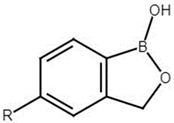	**Compound**	**R Group**
	1-hydroxy-3H-2,1-benzoxaborole	-H
	Tavaborole	-F
	Crisaborole	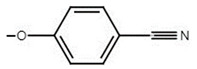

## Data Availability

All data generated or analysed during this study are included in this published article and its Supplementary Materials.

## Supplementary Materials

The following supporting information can be downloaded at: https://www.mdpi.com/article/10.3390/antibiotics15050437/s1, Figure S1: Fluorescence microscopy of *E. coli* BL21(DE3) (DAPI staining, 40×) treated with 1-hydroxy-3H-2,1-benzoxaborole. Figure S2: Fluorescence microscopy of *E. coli* BL21(DE3) (DAPI staining, 40×) treated with 1-hydroxy-3H-2,1-benzoxaborole. Figure S3: Fluorescence microscopy of *E. coli* BL21(DE3) (DAPI staining, 40×) treated with tavaborole. Figure S4: Fluorescence microscopy of *E. coli* BL21(DE3) (DAPI staining, 40×) treated with tavaborole. Figure S5: Fluorescence microscopy of *E. coli* BL21(DE3) (DAPI staining, 40×) treated with crisaborole. Figure S6: Fluorescence microscopy of *E. coli* BL21(DE3) (DAPI staining, 40×) treated with crisaborole. Figure S7: Biofilm eradication assay on *E. coli* ATCC^®^ 25922 biofilms following treatment with 1-hydroxy-3H-2,1-benzoxaborole (a), tavaborole (b), and crisaborole (c). Table S1: Percentage of filament length reduction for 1-hydroxy-3H-2,1-benzoxaborole. Table S2: Percentage of filament length reduction for tavaborole. Table S3: Percentage of filament length reduction for crisaborole. Table S4: Percentage of reduction in biofilm formation for 1-hydroxy-3H-2,1-benzoxaborole. Table S5: Percentage of reduction in biofilm formation for tavaborole. Table S6: Percentage of reduction in biofilm formation for crisaborole. Table S7: Docking scores and MM-GBSA binding free energies of benzoxaborole derivatives in complex with LexA.

## Abbreviations

The following abbreviations are used in this manuscript:

AMR	Antimicrobial resistance
HGT	Horizontal gene transfer
MDR	Multidrug-resistant
CTD	C-terminal domain
NTD	N-terminal domain
LeuRS	Leucyl-tRNA synthetase
PDE4	Phosphodiesterase-4
MIC	Minimum Inhibitory Concentration
