# Supplementary material for: Benzoxaborole-Based Inhibitors Block LexA Autocleavage and Suppress SOS-Dependent Adaptive Phenotypes in Escherichia coli
Source: Antibiotics (Basel). 2026 Apr 27;15(5):437. doi: 10.3390/antibiotics15050437 (PMC13203515; doi:10.3390/antibiotics15050437)
Supplement: Supplementary file 1 [file antibiotics-15-00437-s001.zip › antibiotics-4253815-supplementary.pdf]

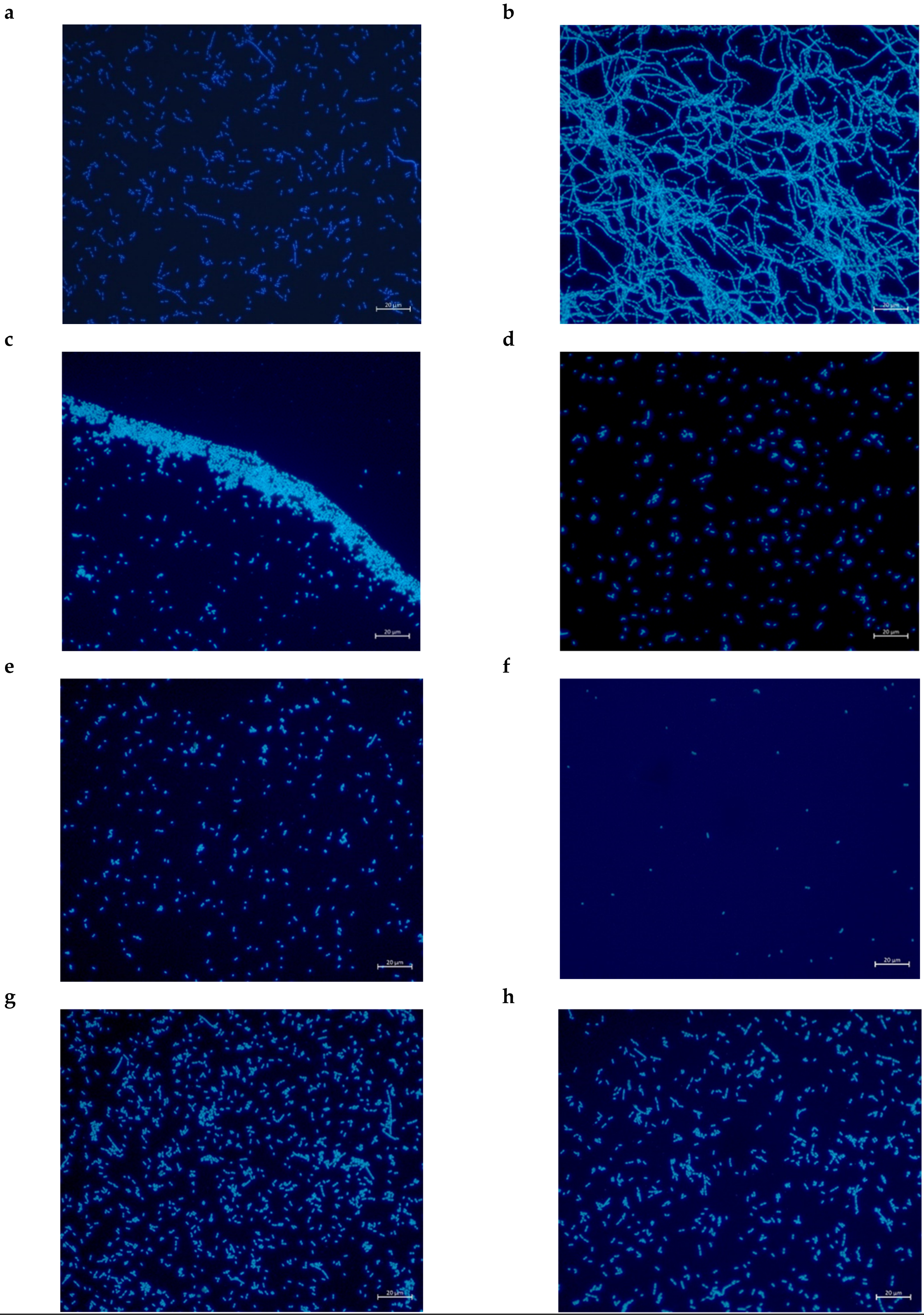

Figure S1. Fluorescence microscopy of *E. coli* BL21(DE3) (DAPI staining, 40×) treated with 1-hydroxy-3H-2,1-benzoxaborole. **(a)** Untreated control; **(b)** levofloxacin (1.9×10^{-3} µg/mL); **(c)** 1-hydroxy-3H-2,1-benzoxaborole (16 µg/mL); **(d)** 1-hydroxy-3H-2,1-benzoxaborole (16 µg/mL) + levofloxacin (1.9×10^{-3} µg/mL); **(e)** 1-hydroxy-3H-2,1-benzoxaborole (8 µg/mL); **(f)** 1-hydroxy-3H-2,1-benzoxaborole (8 µg/mL) +

levofloxacin (1.9×10^{-3} μg/mL); **(g)** 1-hydroxy-3H-2,1-benzoxaborole (4 μg/mL); **(h)** 1-hydroxy-3H-2,1-benzoxaborole (4 μg/mL) + levofloxacin (1.9×10^{-3} μg/mL).

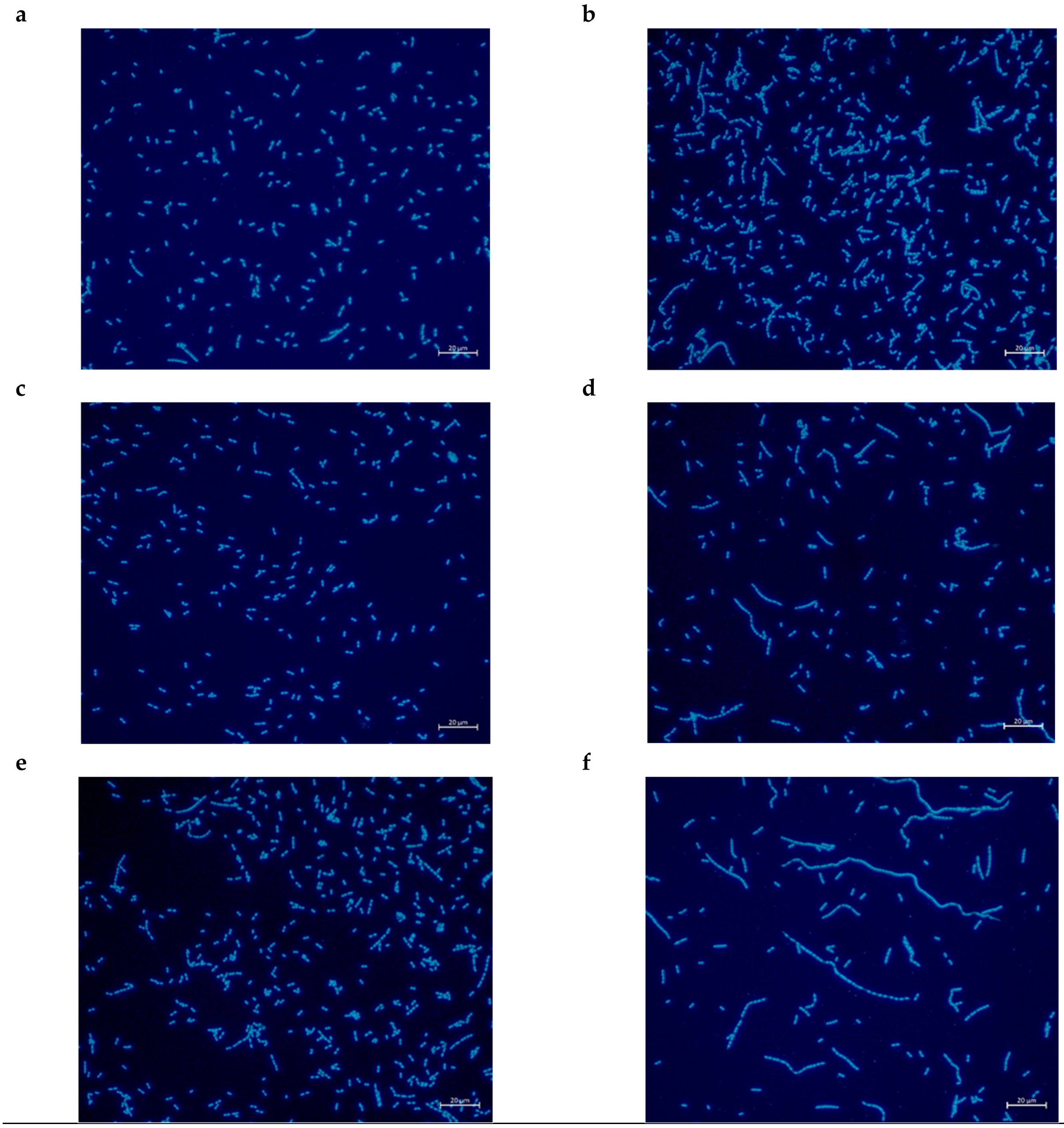

Figure S2. Fluorescence microscopy of *E. coli* BL21(DE3) (DAPI staining, 40×) treated with 1-hydroxy-3H-2,1-benzoxaborole. **(a)** 1-hydroxy-3H-2,1-benzoxaborole (2 μg/mL); **(b)** 1-hydroxy-3H-2,1-benzoxaborole (2 μg/mL) + levofloxacin (1.9×10^{-3} μg/mL); **(c)** 1-hydroxy-3H-2,1-benzoxaborole (1 μg/mL); **(d)** 1-hydroxy-3H-2,1-benzoxaborole (1 μg/mL) + levofloxacin (1.9×10^{-3} μg/mL); **(e)** 1-hydroxy-3H-2,1-benzoxaborole (0.5 μg/mL); **(f)** 1-hydroxy-3H-2,1-benzoxaborole (0.5 μg/mL) + levofloxacin (1.9×10^{-3} μg/mL).

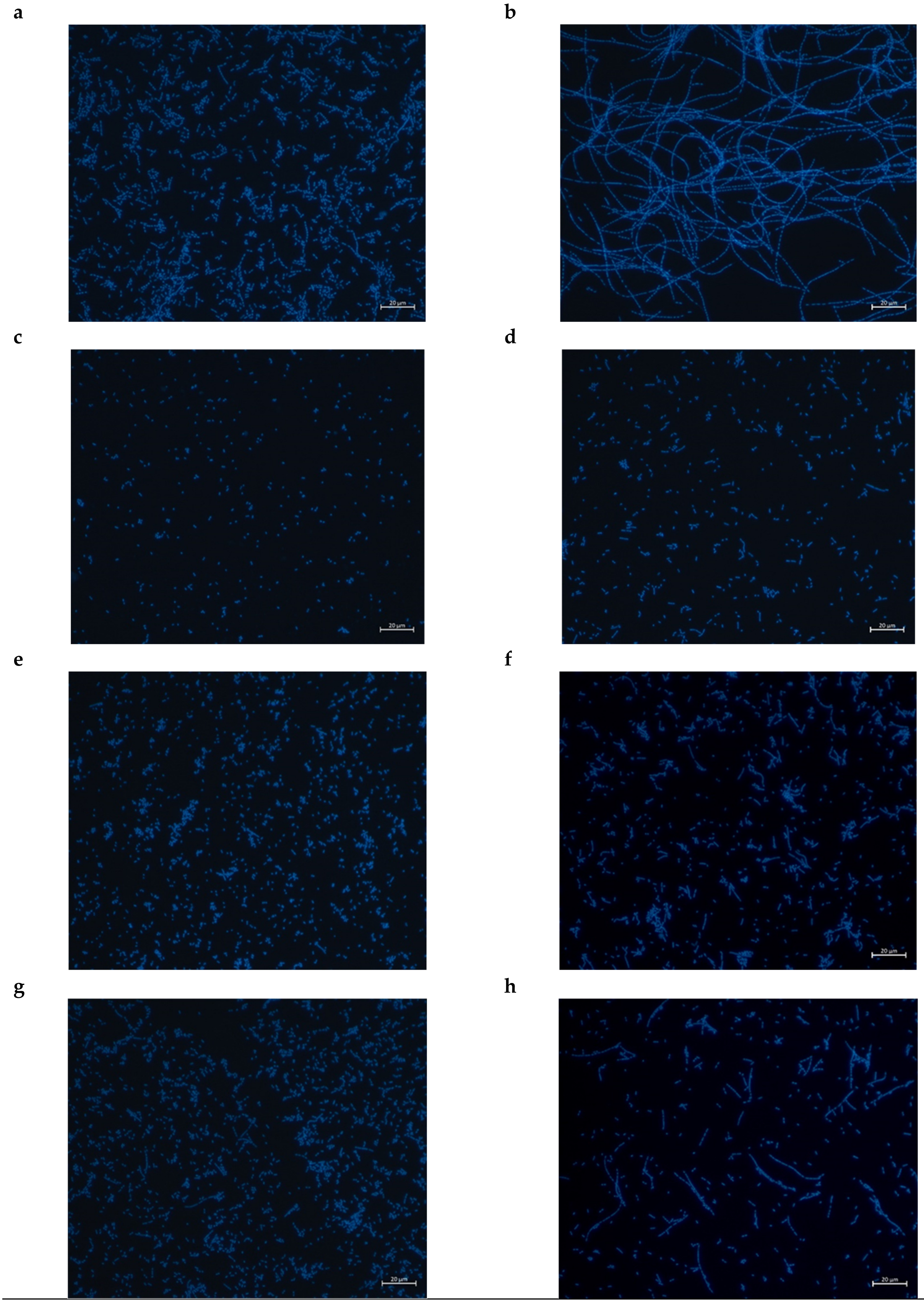

Figure S3. Fluorescence microscopy of *E. coli* BL21(DE3) (DAPI staining, 40×) treated with tavaborole. **(a)** Untreated control; **(b)** levofloxacin (1.9×10^{-3} μg/mL); **(c)** tavaborole (8 μg/mL); **(d)** tavaborole (8 μg/mL) + levofloxacin (1.9×10^{-3} μg/mL); **(e)** tavaborole (4 μg/mL); **(f)** tavaborole (4 μg/mL) + levofloxacin (1.9×10^{-3} μg/mL); **(g)** tavaborole (2 μg/mL); **(h)** tavaborole (2 μg/mL) + levofloxacin (1.9×10^{-3} μg/mL).

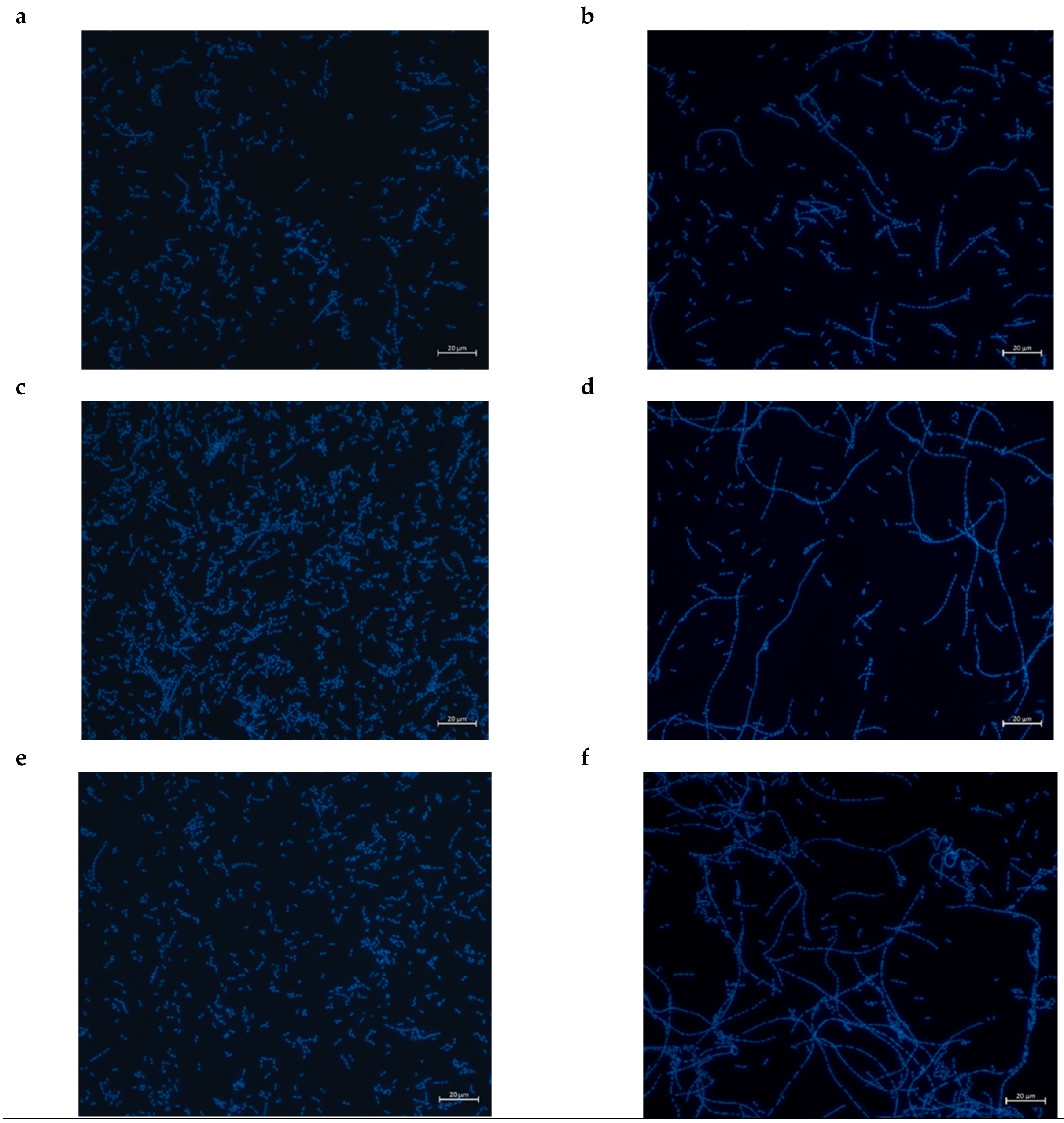

Figure S4. Fluorescence microscopy of *E. coli* BL21(DE3) (DAPI staining, 40×) treated with tavaborole. **(a)** tavaborole (1 µg/mL); **(b)** tavaborole (1 µg/mL) + levofloxacin (1.9×10^{-3} µg/mL); **(c)** tavaborole (0.5 µg/mL); **(d)** tavaborole (0.5 µg/mL) + levofloxacin (1.9×10^{-3} µg/mL); **(e)** tavaborole (0.25 µg/mL); **(f)** tavaborole (0.25 µg/mL) + levofloxacin (1.9×10^{-3} µg/mL).

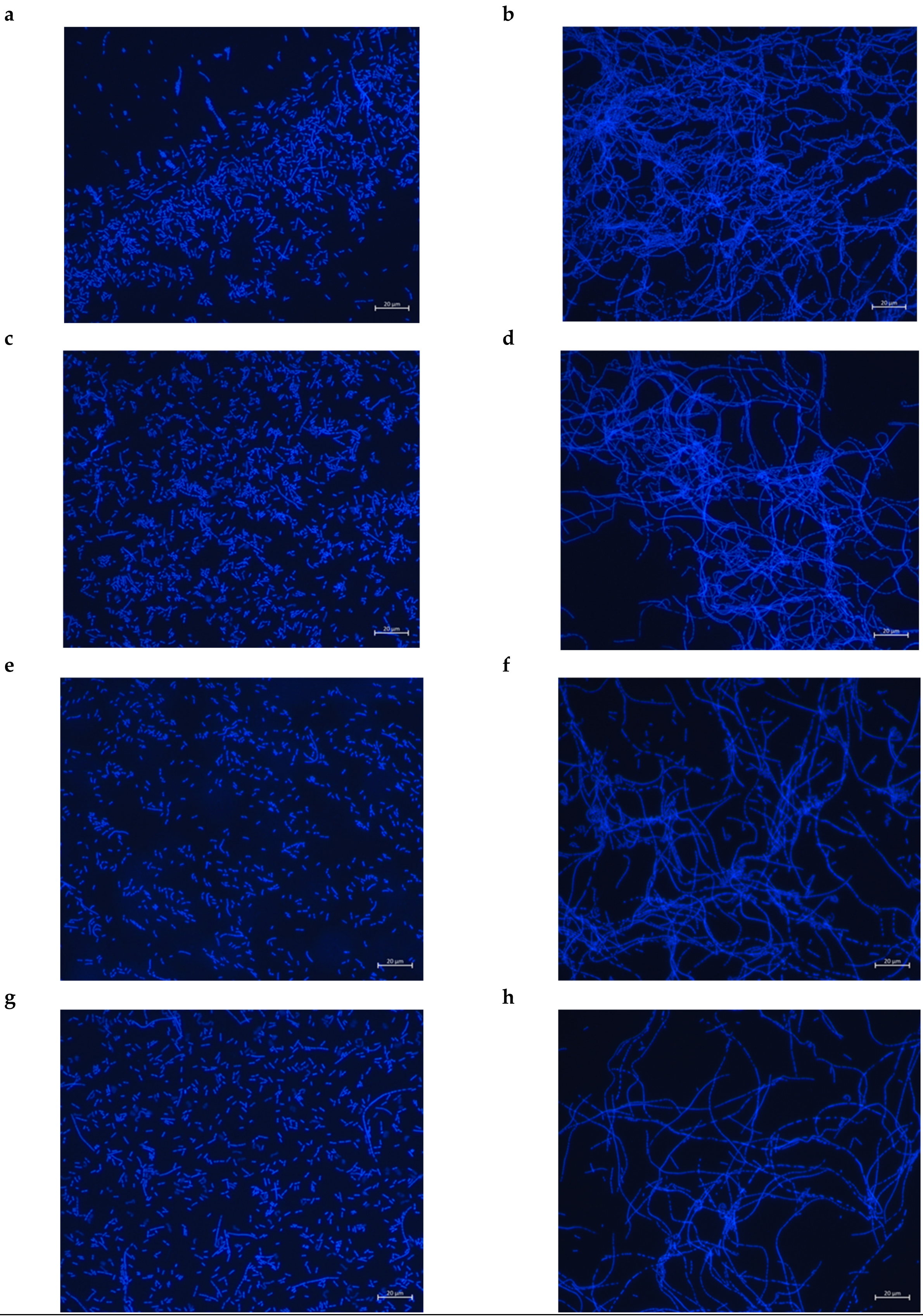

Figure S5. Fluorescence microscopy of *E. coli* BL21(DE3) (DAPI staining, 40×) treated with crisaborole. **(a)** Untreated control; **(b)** levofloxacin (1.9×10^{-3} μg/mL); **(c)** crisaborole (128 μg/mL); **(d)** crisaborole (128 μg/mL) + levofloxacin (1.9×10^{-3} μg/mL); **(e)** crisaborole (64 μg/mL); **(f)** crisaborole (64 μg/mL) + levofloxacin (1.9×10^{-3} μg/mL); **(g)** crisaborole (32 μg/mL); **(h)** crisaborole (32 μg/mL) + levofloxacin (1.9×10^{-3} μg/mL).

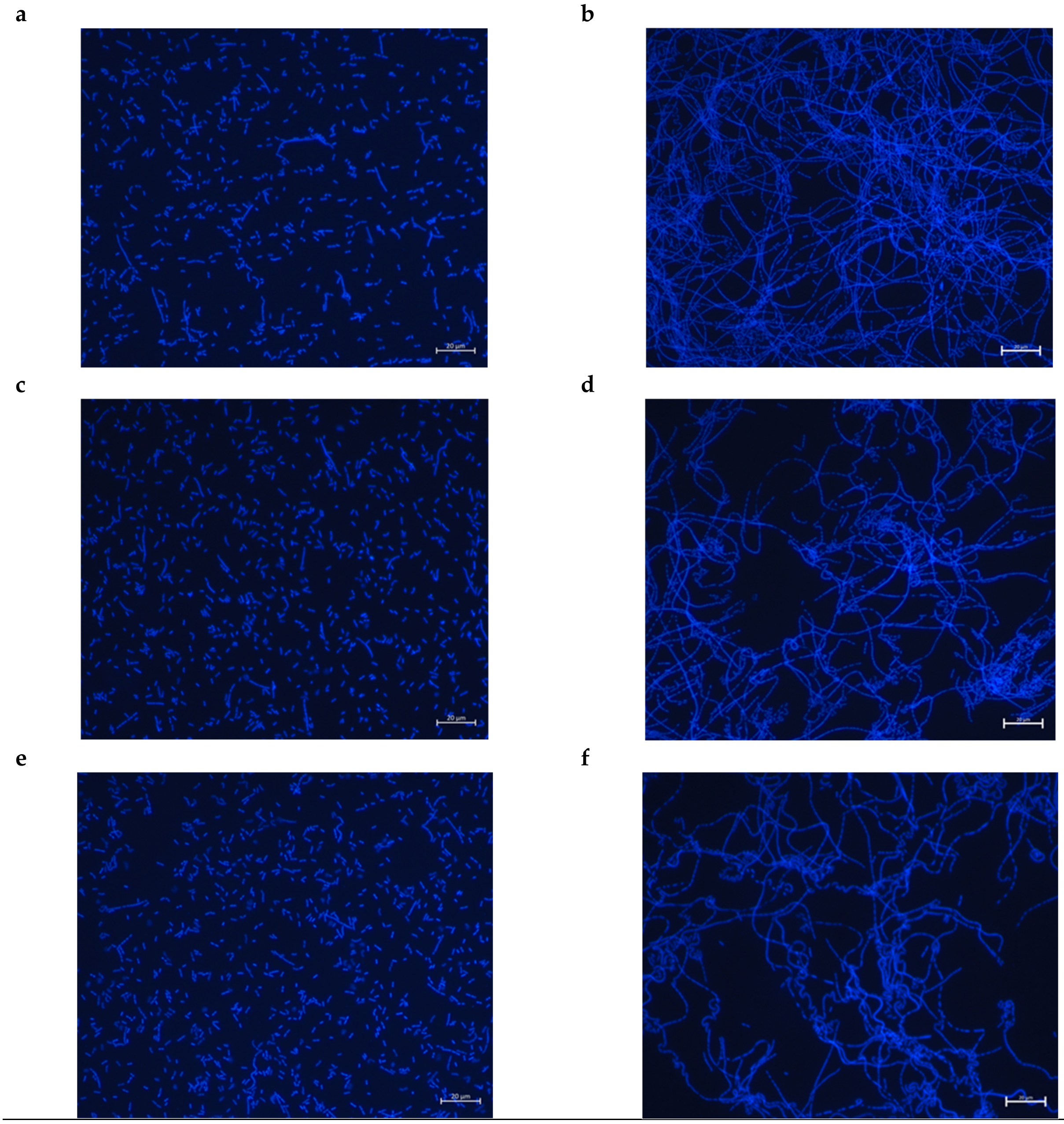

Figure S6. Fluorescence microscopy of *E. coli* BL21(DE3) (DAPI staining, 40×) treated with crisaborole. **(a)** crisaborole (16 μg/mL); **(b)** crisaborole (16 μg/mL) + levofloxacin (1.9×10^{-3} μg/mL); **(c)** crisaborole (8 μg/mL); **(d)** crisaborole (8 μg/mL) + levofloxacin (1.9×10^{-3} μg/mL); **(e)** crisaborole (4 μg/mL); **(f)** crisaborole (4 μg/mL) + levofloxacin (1.9×10^{-3} μg/mL).

Table S1. Percentage of filament length reduction for 1-hydroxy-3H-2,1-benzoxaborole.

Compound(s) (μg/mL)	Filament length (μm)	SD	Percentage of reduction
LVX (1.9×10^{-3})	207.29	59.06	-
LVX+ 1-hydroxy-3H-2,1-benzoxaborole (0.5)	61.97	51.19	70%
LVX + 1-hydroxy-3H-2,1-benzoxaborole (1)	40.01	42.68	81%
LVX + 1-hydroxy-3H-2,1-benzoxaborole (2)	27.89	16.97	87%
LVX + 1-hydroxy-3H-2,1-benzoxaborole (4)	18.75	14.29	91%
LVX + 1-hydroxy-3H-2,1-benzoxaborole (8)	8.76	6.26	96%
LVX + 1-hydroxy-3H-2,1-benzoxaborole (16)[#]	6.59	3.48	97%

[#] MIC value

Table S2. Percentage of filament length reduction for tavaborole.

Compound(s) (μg/mL)	Filament length (μm)	SD	Percentage of reduction
LVX (1.9×10^{-3})	212.06	72.58	-
LVX+ tavaborole (0.25)	143.26	79.92	32%
LVX + tavaborole (0.5)	140.3	63.63	34%
LVX + tavaborole (1)	66.88	46.49	68%
LVX + tavaborole (2)	37.39	23.19	82%
LVX + tavaborole (4)	15.71	11.81	93%
LVX + tavaborole (8)[#]	6.1	4.39	97%

[#] MIC value

Table S3. Percentage of filament length reduction for crisaborole.

Compound(s) (μg/mL)	Filament length (μm)	SD	Percentage of reduction
LVX (1.9×10^{-3})	202.9	109.27	-
LVX+ crisaborole (4)	188.6	81.47	7%
LVX + crisaborole (8)	181.8	82.49	10%
LVX + crisaborole (16)	183.41	85.45	10%
LVX + crisaborole (32)	151.12	94.33	26%
LVX + crisaborole (64)	127.05	79.91	37%
LVX + crisaborole (128)[#]	93.58	69.45	54%

[#] The MIC value is higher than 128 μg/mL

Table S4. Percentage of reduction of biofilm formation for 1-hydroxy-3H-2,1-benzoxaborole.

Compound concentration (µg/mL)	Biofilm biomass (OD_{595nm})	SD	Percentage of reduction
0	1.13637	0.06438	-
128	0.13117	0.00915	88%
64	0.1303	0.00924	89%
32	0.17327	0.03569	85%
16	0.39235	0.08591	65%
8[#]	1.05042	0.15391	8%
4	1.4554	0.22292	-28%
2	1.57638	0.25092	-39%
1	1.52815	0.23336	-34%
0.5	1.47003	0.24699	-29%
0.25	1.25855	0.07576	-11%
0.125	1.26683	0.08569	-11%
0.0625	1.18145	0.07131	-4%

[#] MIC value

Table S5. Percentage of reduction of biofilm formation for tavaborole.

Compound concentration (µg/mL)	Biofilm biomass (OD_{595nm})	SD	Percentage of reduction
0	1.22433	0.0526	-
128	0.12298	0.00844	90%
64	0.1621	0.02146	87%
32	0.1827	0.03078	85%
16	0.21293	0.03742	83%
8[#]	0.32948	0.06034	73%
4	0.64717	0.0988	47%
2	1.45342	0.20341	-19%
1	1.53717	0.23208	-26%
0.5	1.43752	0.10121	-17%
0.25	1.4338	0.12821	-17%
0.125	1.3571	0.09654	-11%
0.0625	1.22655	0.05946	0%

[#] MIC value

Table S6. Percentage of reduction of biofilm formation for crisaborole.

Compound concentration (µg/mL)	Biofilm biomass (OD_{595nm})	SD	Percentage of reduction
0	1.06574	0.32841	-
128#	1.14153	0.05606	-7%
64	1.22877	0.04373	-15%
32	1.31055	0.06192	-23%
16	1.35645	0.08735	-27%
8	1.51575	0.23847	-42%
4	1.59282	0.29011	-49%
2	1.51643	0.28044	-42%
1	1.52947	0.27106	-44%
0.5	1.2465	0.15864	-17%
0.25	1.1487	0.05135	-8%
0.125	1.13553	0.05099	-7%
0.0625	1.0842	0.03585	-2%

The MIC value is higher than 128 µg/mL

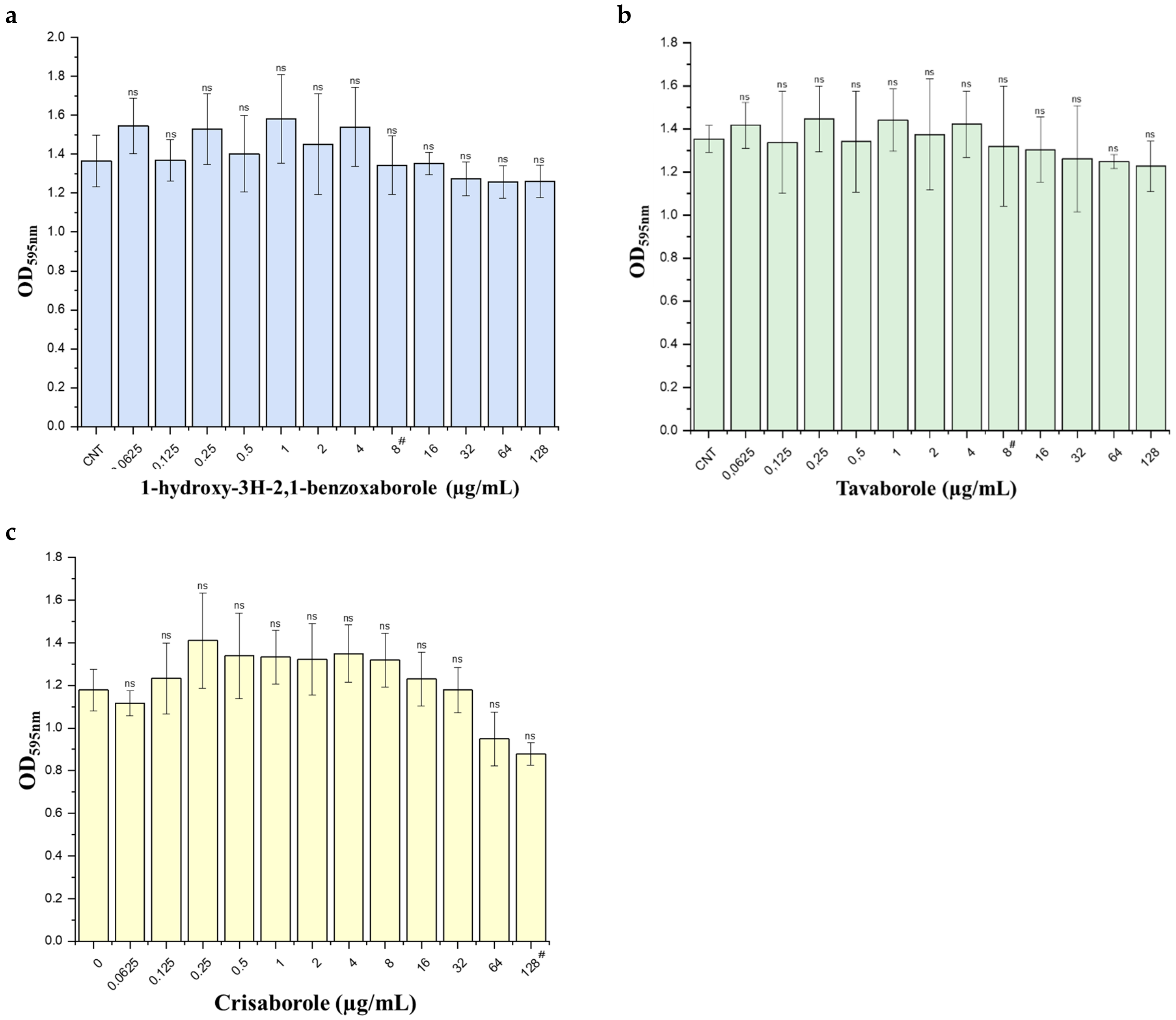

Figure S7. Biofilm eradication assay on *E. coli* ATCC® 25922 biofilms following treatment with 1-hydroxy-3H-2,1-benzoxaborole **(a)**, tavaborole **(b)**, and crisaborole **(c)**. # MIC value; (ns) not significant.

Table S7. Docking scores and MM-GBSA binding free energies of benzoxaborole derivatives in complex with LexA

**Compound(s)**	**Docking score (kcal/mol)**	**MM-GBSA (kcal/mol)**
1-hydroxy-3H-2,1-benzoxaborole	-4.154 (upward)<br>-3.954 (downward)	-27.80 (upward)<br>-9.68 (downward)
Tavaborole	-5.053	-25.88
Crisaborole	-3.820	-41.03
